# QTL mapping and candidate gene analysis of peduncle vascular bundle related traits in rice by genome-wide association study

**DOI:** 10.1186/s12284-018-0204-7

**Published:** 2018-03-06

**Authors:** Laiyuan Zhai, Tianqing Zheng, Xinyu Wang, Yun Wang, Kai Chen, Shu Wang, Yun Wang, Jianlong Xu, Zhikang Li

**Affiliations:** 1 Rice Research Institute, Shenyang Agricultural University/Key Laboratory of Northern Japonica Rice Genetics and Breeding, Ministry of Education, Shenyang, 110866 China; 2 Institute of Crop Sciences/National Key Facility for Crop Gene Resources and Genetic Improvement, Chinese Academy of Agricultural Sciences, 12# South Zhong-Guan-Cun Street, Haidain District, Beijing, 100081 China; 3 Agricultrual Genomics Institute at Shenzhen, Chinese Academy of Agricultural Sceinces, Shenzhen, 518120 China; 4 Shenzhen Institute of Breeding and Innovation, Chinese Academy of Agricultural Sciences, Shenzhen, 518120 China

**Keywords:** GWAS, Vascular bundle, SNPs, Candidate genes, Quantitative trait loci/locus (QTL)

## Abstract

**Background:**

The vascular bundle especially in the peduncle is one of crucial limiting factors of rice yield, and it determines how plants efficiently transport photosynthetic products, mineral nutrients and water from leaf and root to panicle. However, the genetic base of rice vascular bundle related traits in the peduncle still remains unknown.

**Results:**

The 423 panel showed substantial natural variations of peduncle vascular bundle. In total, 48 quantitative trait loci/locus (QTL) affecting the eight traits were identified throughout the genome by applying a significance threshold of *P* < 1.0 × 10^− 4^. Combined determining linkage disequilibrium (LD) blocks associated with significant SNPs and haplotype analyses allowed us to shortlist six candidate genes for four important QTL regions affecting the peduncle vascular bundle traits, including one cloned gene (*NAL1*) and three newly identified QTL (*qLVN6*, *qSVN7,* and *qSVA8.1*). Further the most likely candidate genes for each important QTL were also discussed based on functional annotation.

**Conclusions:**

Genetic base on peduncle vascular bundle related traits in rice was systematically dissected, and most likely candidate genes of the known gene *NAL1* and the three newly identified QTL (*qLVN6*, *qSVN7,* and *qSVA8.1*) were analyzed. The results provided valuable information for future functional characterization and rice breeding for high yield through optimizing transportation efficiency of photosynthetic products by marker-assisted selection.

**Electronic supplementary material:**

The online version of this article (10.1186/s12284-018-0204-7) contains supplementary material, which is available to authorized users.

## Background

Rice (*Oryza sativa* L.) is one of the most important cereal crops and a staple food for more than one-half of the world’s population. In retrospect of rice breeding history, good harmony of source, sink and translocation capacity (i.e. flow) of assimilates plays an important role in improvement of rice yield potential (Donald 1968; Lafitte and Travis 1984; Ashraf et al. 1994). Among them, three top leaves especially flag leaf are main source traits of assimilate synthesis while spikelets including grain number and size are main traits of accumulation of assimilate. The vascular bundle especially in the peduncle is the transport system that links source and sink, and it determines how plants efficiently transport photosynthetic products, mineral nutrients and water from source to sink (Housley and Peterson 1982). Rice breeding history indicated that good balance among source, sink and flow of assimilates, namely, large sink, sufficient source and flow fluency is prerequisite for high yield potential of rice (Ashraf et al. 1994).

The vascular bundle systems in plants play a significant role in delivering water and nutrients as well as other substances needed for growth and defense (Lucas et al. 2013). Vascular bundles, which interconnect all parts of the plant, extending from the stem into leaves, and down into the root system. It consists of xylem and phloem, two differentiated conductive tissues, as well as undifferentiated cambial or procambial stem cells. Xylem transports water and dissolved minerals, whereas phloem is required for the distribution of mainly photosynthetic products (sugars, RNA, proteins, and other organic compounds) from source to sink organs (Dettmer et al. 2009). A significant positive correlation between grain yield and the number of vascular bundles and the area of phloem in peduncle have been reported in rice (Ashraf et al. 1994), wheat (Evans et al. 1970) and oat (Housley and Peterson. 1982). The capacity of the vascular bundle system for efficiently transporting assimilates from the source to the sink has been shown as a limiting factor for yield potential realization (Housley and Peterson 1982). Additionally, the differentiation in vascular bundle system is an important parameter that defines differentiation between *indica* and *japonica* rice (Fukuyama et al. 1996). Generally, *indica* varieties tend to have more number of large vascular bundle (LVN) than *japonica* varieties, though there is no significant difference in the number of small vascular bundle (SVN) between them. The exploitation of the great heterosis that exists in the inter-subspecific crosses between *indica* and *japonica* rice has long been considered as a promising way to increase the yield potential. It is therefore of importance to study the genetic basis of peduncle vascular bundle to provide useful information for the genetic improvement of rice yield potential using heterosis between *indica* and *japonica*.

The use of QTL mapping has contributed to a better understanding of the genetic basis of many agronomic important traits. For past decades, many researchers have identified QTL for peduncle vascular bundle related traits in rice (Sasahara et al. 1999; Zhang et al. 2002; Cui et al. 2003; Bai et al. 2012). Notably, several QTL affecting the vascular bundle system in rice have been further cloned. For instance, genes such as *APO1* (Terao et al. 2010), *AVB* (Ma et al. 2017) and *NAL1* (Qi et al. 2008; Fujita et al. 2013) have been reported to involve in enhanced translocation capacity of vascular bundles.

Cloning QTL affecting complex traits has been a major challenge to plant geneticists and molecular biologists since the classical strategy using map-based cloning of QTL is extremely troublesome and time-consuming. In recent years, GWAS, based on the genome sequencing and SNP chip technology, makes large-scale high-precision identification to the alleles and the variation which are widely distributed in natural groups of rice become reality (Huang et al. 2010; Zhao et al. 2011; Han and Huang. 2013). In the present study, we reported the first GWAS for vascular bundle system in any agricultural crop using a panel of germplasm resources with a broad genetic diversity. A diverse panel consisting of 423 accessions selected from 3 K Rice Genome Project (3 K RGP) (3K RGP 2014; Sun et al. 2017) was evaluated in 2015 and 2016. GWAS was performed using 27 K SNPs generated from 3 K RGP through high-throughput sequencing technologies (Zheng et al. 2015). And then the candidate genes based on the SNPs significantly correlated to the vascular bundles through GWAS can be identified.

## Results

### Trait variance and correlations

**Fig. 1:**
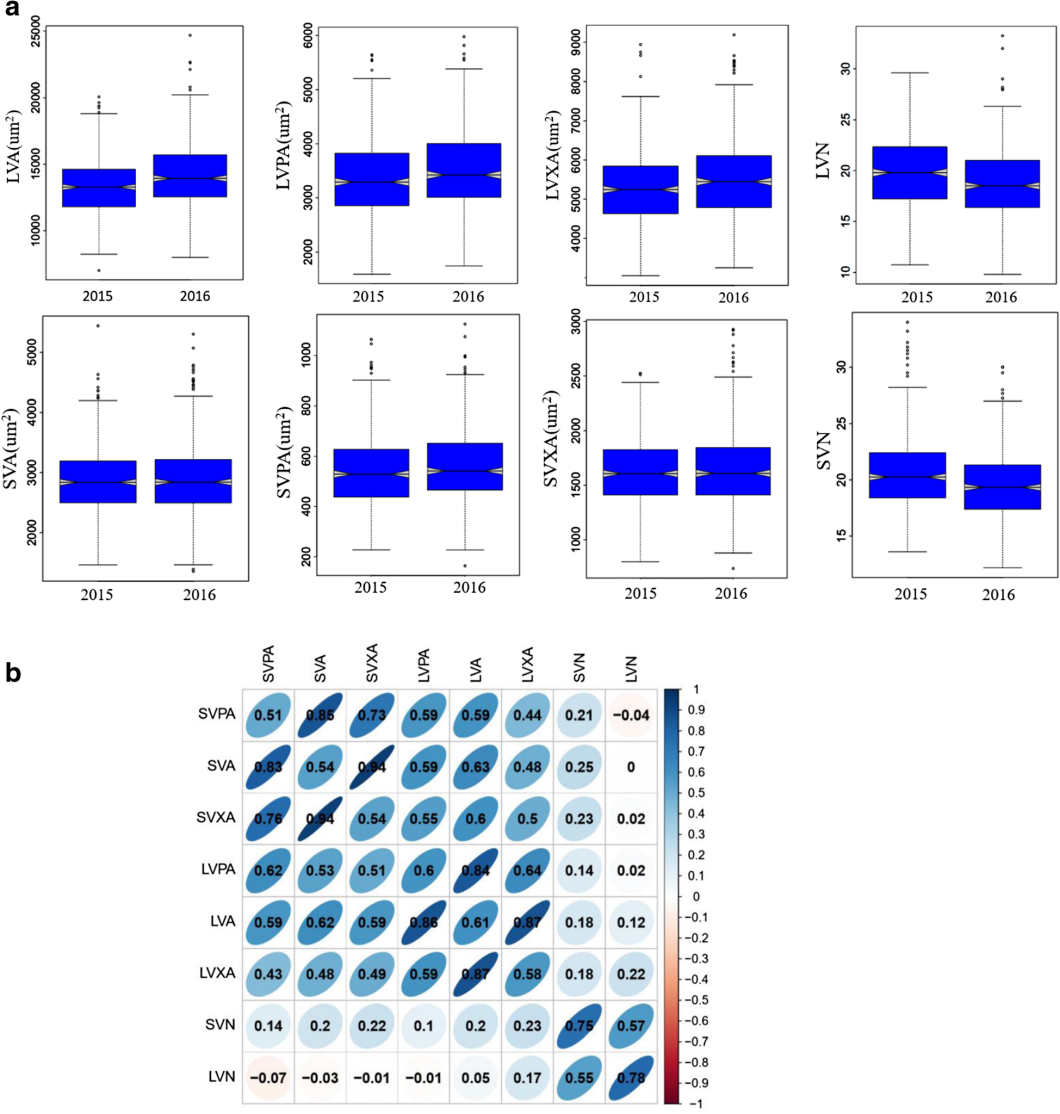
**a** Box plots of eight rice vascular bundle related traits; the number of large vascular bundle (LVN), the area of large vascular bundle (LVA), the xylem area of large vascular bundle (LVXA), the phloem area of large vascular bundle (LVPA), the number of small vascular bundle (SVN), the area of small vascular bundle (SVA), the phloem area of small vascular bundle (SVPA) and the xylem area of small vascular bundle (SVXA). **b** Correlations between eight evaluated traits in 2015 (upper triangular) and 2016 (lower triangular). The values on principal diagonal indicate correlations between 2015 and 2016. The areas and colors of ellipses indicate the absolute value of corresponding r. Right and left oblique ellipses indicate positive and negative correlations, respectively

In general, most of the vascular bundle related traits in peduncle appeared to be normally distributed, but a few traits, phloem area of large vascular bundle (LVPA) and phloem area of small vascular bundle (SVPA) showed skewed distributions in 2016 (Fig. 1a). The panel presented substantial variations for all the measured traits. The phenotype pairwise correlations between the measured traits were similar in both years. In 2015 and 2016, strong positive correlations were observed for all area related traits (total area, phloem area, and xylem area) between large and small vascular bundle, indicating the consistent effects of vascular bundle on these traits across all accessions. As expected, the vascular bundle total area had significant positive correlations with its corresponding component traits (phloem area and xylem area) whereas no significant correlations were detected between the vascular bundle total area and the number of vascular bundle (Fig. 1b).

### Basic statistics of markers

For the 26,097 high quality SNPs, the number of markers per chromosome ranged from 1600 on chromosome 9 to 3101 on chromosome 1. The size of chromosome varied from 22.93 Mb for chromosome 9 to 43.25 Mb for chromosome 1. The whole genome size was 372.50 Mb. The average marker spacing was 14.27 kb with spacing ranging from 12.68 kb for chromosome 10 to 15.50 kb for chromosome 4 (Additional file 1: Figure S1).

### Population structure

**Fig. 2:**
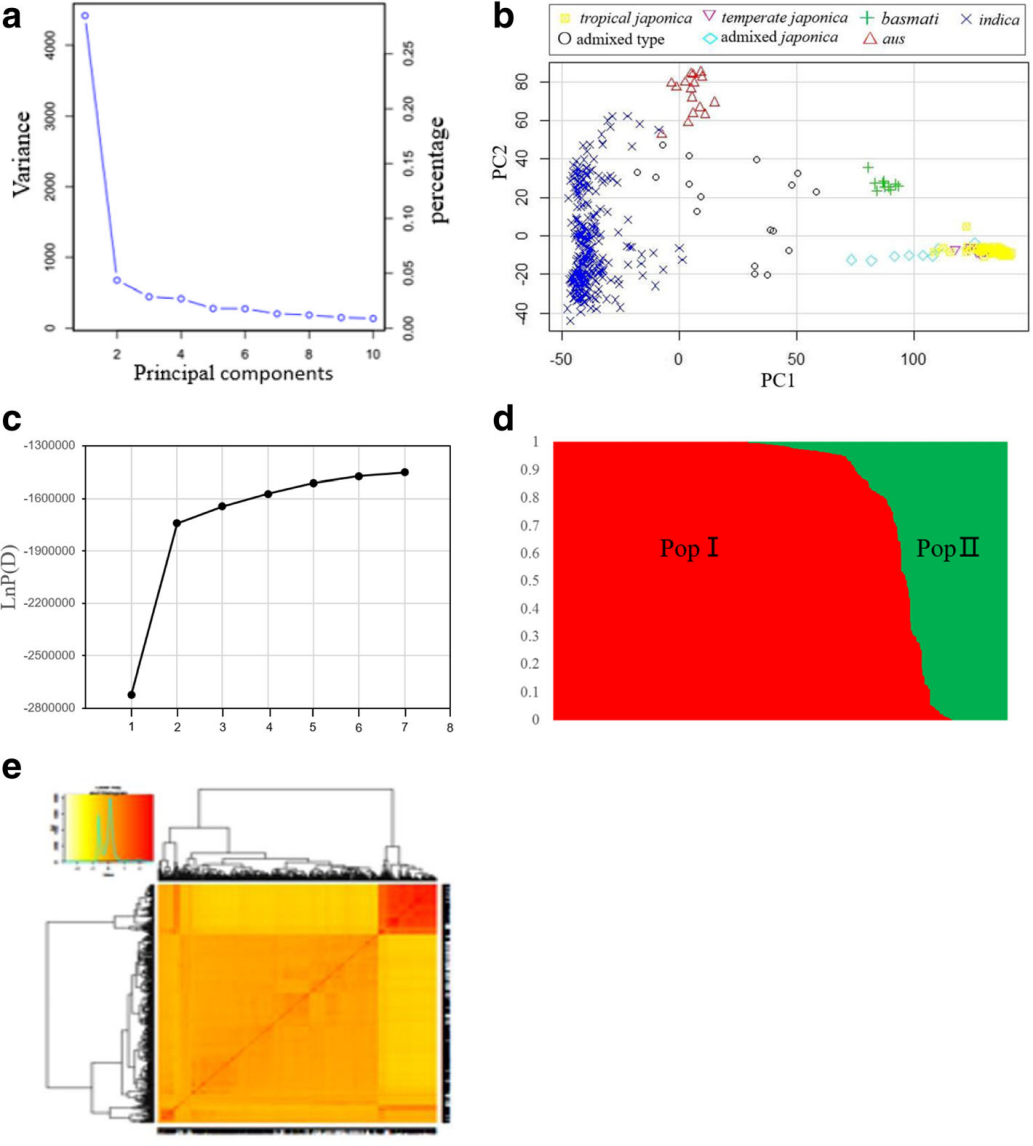
**a** Screen plot from GAPIT showing the selection of PCs for association study. **b** PCA plot based on the screen plot in the rice diversity panel. **c** Screen plot from STRUCTURE showing the selection of Q. **d** Bayesian clustering of 423 accessions using STRUCTURE program. **e** Heat map of kinship from GAPIT with the tree shown on the top and left

The screen plot showed that two principal component (PC) can be reserved as informative (Fig. 2a–b; Additional file 2: Table S1), where downhill changed gradually (Fig. 2a). The score plot of PC showed the distribution of each accession in the rice diversity panel (Fig. 2b). Additionally, the highest log likelihood scores of the population structure were observed when the number of populations was set at 2 (K = 2; Fig. 2c), indicating that there were two distinct subpopulations (Pop I and Pop II) in the current panel (Fig. 2d; Additional file 2: Table S1). And the heat map of kinship relatedness matrix (KI) showed the same result (Fig. 2e).

### Detection of QTL by GWAS

A total of 48 different QTL for all traits were identified in 2015 and 2016, ranging from two QTL for SVPA to 10 QTL for LVPA. Among them, 23 (37) QTL were detected in 2015 (2016) and 12 QTL were commonly detected in two years (Table 1; Additional file 3: Figure S2).

**Table 1:** QTL identified for eight peduncle vascular bundle related traits in two years

Trait^a^	QTL	Year	Peak SNP	Allele^b^	*P*	Effect^c^	R^2^ (%)^d^
LVN	*qLVN1*	2016	Chr1-13,801,593	T/A	7.8E-05	−0.82	2.4
	*qLVN2.1*	2015	Chr2–31,993	A/G	4.8E-05	−0.89	2.4
	*qLVN2.2*	2016	Chr2–25,871,069	A/G	5.5E-05	1.64	2.5
	*qLVN2.3*	2016	Chr2–29,521,254	T/C	8.8E-05	−1.56	2.2
	*qLVN4*	2015	Chr4–5,193,957	G/A	8.1E-05	0.77	2.2
	*qLVN6*	2015	Chr6–28,662,981	C/T	9.4E-05	−0.67	2.0
		2016	Chr6–28,639,502	G/C	9.5E-05	−0.51	2.4
	*qLVN11*	2016	Chr11–1,063,214	C/T	1.3E-05	1.37	3.0
LVA	*qLVA1*	2016	Chr1–2,179,066	A/G	4.4E-05	− 617.1	3.8
	*qLVA3*	2016	Chr3–33,078,718	T/C	8.2E-05	− 714.3	3.5
	*qLVA4.1*	2015	Chr4–31,081,174	C/A	9.6E-05	− 392.1	1.4
		2016	Chr4–31,068,550	G/C	4.1E-05	− 1073.3	3.8
	*qLVA4.2*	2016	Chr4–32,189,616	T/C	3.8E-05	− 960.8	3.9
	*qLVA6*	2015	Chr6–20,057,366	C/T	6.1E-05	1362.6	3.4
	*qLVA8*	2015	Chr8–3770	G/A	6.4E-05	− 524.4	2.5
		2016	Chr8–69,600	C/T	6.4E-05	− 620.0	3.6
	*qLVA11*	2015	Chr11–21,798,323	T/G	7.1E-05	463.6	3.0
		2016	Chr11–21,798,323	T/G	9.0E-05	582.6	3.4
LVPA	*qLVPA1.1*	2016	Chr1–1,806,093	C/A	6.4E-05	252.9	3.5
	*qLVPA1.2*	2016	Chr1–2,179,066	A/G	8.7E-05	− 175.1	3.4
	*qLVPA2*	2016	Chr2–30,012,958	C/G	8.0E-05	− 508.7	3.4
	*qLVPA4.1*	2015	Chr4–31,081,174	C/A	6.7E-05	−83.1	3.0
		2016	Chr4–31,068,550	G/C	3.0E-05	− 322.3	3.9
	*qLVPA4.2*	2016	Chr4–32,189,616	T/C	6.1E-05	−277.5	3.6
	*qLVPA8.1*	2016	Chr8–3770	G/A	4.5E-05	− 219.5	3.7
	*qLVPA8.2*	2015	Chr8–2,393,328	T/A	3.8E-05	−524.5	3.6
	*qLVPA8.3*	2015	Chr8–27,101,483	C/T	9.5E-05	220.4	3.2
	*qLVPA8.4*	2015	Chr8–27,737,011	C/T	2.9E-05	187.6	3.7
		2016	Chr8–27,707,779	C/T	9.2E-05	−190.4	2.8
	*qLVPA11*	2015	Chr11–25,611,048	C/A	3.9E-05	−360.2	3.5
LVXA	*qLVXA1*	2016	Chr1–2,758,104	A/G	5.8E-05	− 514.4	3.7
	*qLVXA5*	2015	Chr5–4,823,527	C/T	8.4E-05	− 224.9	3.2
		2016	Chr5–4,823,527	C/T	3.4E-05	− 287.8	3.9
	*qLVXA6*	2016	Chr6–21,044,730	C/A	5.9E-05	415.6	3.7
	*qLVXA8.1*	2015	Chr8–87,741	T/C	5.4E-05	220.6	2.8
		2016	Chr8–69,600	C/T	1.4E-06	− 325.9	5.3
	*qLVXA8.2*	2015	Chr8–220,938	C/T	3.0E-05	195.8	2.3
		2016	Chr8–220,938	C/T	2.7E-05	282.4	4.0
	*qLVXA8.3*	2015	Chr8–27,031,242	T/A	8.6E-05	439.9	3.2
	*qLVXA11*	2015	Chr11–21,798,323	T/G	1.6E-05	245.6	3.9
		2016	Chr11–21,798,323	T/G	2.7E-05	222.9	2.7
SVN	*qSVN1*	2016	Chr1–9,724,654	T/C	4.3E-05	−1.76	3.6
	*qSVN2*	2016	Chr2–25,915,457	C/A	5.5E-05	−1.98	3.5
	*qSVN5.1*	2015	Chr5–24,874,414	A/G	3.3E-05	−1.69	3.9
		2016	Chr5–24,874,414	A/G	2.7E-06	−1.77	4.7
	*qSVN5.2*	2016	Chr5–25,915,442	G/A	5.7E-05	1.97	3.5
	*qSVN7*	2015	Chr7–29,604,076	C/T	4.7E-05	1.27	2.8
		2016	Chr7–29,604,076	C/T	1.9E-06	1.69	4.9
SVA	*qSVA2.1*	2015	Chr2–10,294,412	G/A	8.0E-05	139.9	3.7
	*qSVA2.2*	2016	Chr2–11,856,232	C/A	3.7E-05	−342.0	3.7
	*qSVA8.1*	2016	Chr8–2,031,279	C/T	3.0E-05	− 258.4	3.8
	*qSVA8.2*	2016	Chr8–20,982,368	G/T	7.9E-05	−251.3	3.4
	*qSVA11*	2015	Chr11–25,611,048	C/A	6.67E-05	−303.8	3.6
	*qSVA12*	2016	Chr12–3,630,490	C/T	5.17E-05	− 234.6	3.5
SVPA	*qSVPA4.1*	2016	Chr4–13,422,276	C/T	6.53E-05	110.0	3.7
	*qSVPA4.2*	2016	Chr4–31,878,154	A/G	3.56E-05	38.7	3.9
SVXA	*qSVXA2*	2016	Chr2–30,012,958	C/G	6.4E-05	−265.8	3.5
	*qSVXA8*	2016	Chr8–2,031,279	C/T	9.74E-05	− 145.4	3.4
	*qSVXA9*	2015	Chr9–16,464,178	C/T	1.28E-05	−149.5	4.4
	*qSVXA11*	2015	Chr11–25,611,048	C/A	1.13E-05	−88.2	4.5

^a^LVN the number of large vascular bundle, LVA the total area of large vascular bundle; LVPA the phloem area of large vascular bundle, LVXA the xylem area of large vascular bundle, SVN the number of small vascular bundle, SVA the total area of small vascular bundle, SVPA the phloem area of small vascular bundle, SVXA the xylem area of small vascular bundle

^b^Major/Minor allele

^c^Effect: Allele effect with respect to the minor allele

^d^R^2^ (%): Phenotypic variance explained

For LVN, seven QTL were detected on chromosomes 1, 2, 4, 6 and 11. Among them, *qLVN2.1* and *qLVN4* were detected in 2015, and accounted for 2.4% and 2.2% of phenotypic variance, respectively. Four QTL, *qLVN1*, *qLVN2.2*, *qLVN2.3* and *qLVN11*, were detected in 2016, and explained 2.2% to 3.0% of phenotypic variance. Only *qLVN6* was detected in both 2015 and 2016, and explained the average phenotypic variance of 2.2% (Table 1; Additional file 3: Figure S2).

For total area of large vascular bundle (LVA), seven QTL were detected on chromosomes 1, 3, 4, 6, 8 and 11. The *qLVA6* with 3.4% of phenotypic variance was only detected in 2015 and three QTL (*qLVA1*, *qLVA3*, and *qLVA4.2*) were only detected in 2016, which were accounted for phenotypic variance from 3.5% to 3.9%. Three QTL, *qLVA4.1*, *qLVA8* and *qLVA11*, were detected in both years and explained the average phenotypic variance of 2.6%, 3.1% and 3.2%, respectively (Table 1; Additional file 3: Figure S2).

Ten QTL for LVPA were detected on chromosomes 1, 2, 4, 8 and 11. Three QTL (*qLVPA8.2*, *qLVPA8.3* and *qLVPA11*) were detected in 2015, and accounted for 3.2% to 3.6% of phenotypic variance. Five QTL (*qLVPA1.1*, *qLVPA1.2*, *qLVPA2*, *qLVPA4.2* and *qLVPA8.1*) were detected in 2016 and accounted for phenotypic variance from 3.4% to 3.7%. Another two QTL, *qLVPA4.1* and *qLVPA8.4* were commonly detected in the two years, and explained the average phenotypic variance 3.5% and 3.3%, respectively (Table 1; Additional file 3: Figure S2).

For xylem area of large vascular bundle (LVXA), seven QTL were detected on chromosomes 1, 5, 6, 8, and 11. Among them, only *qLVXA8.3* was detected in 2015 and explained 3.2% of phenotypic variance. Two QTL, *qLVXA1* and *qLVXA6*, were detected in 2016 and accounted for both 3.7% of phenotypic variance. Four QTL, namely, *qLVXA5*, *qLVXA8.1*, *qLVXA8.2* and *qLVXA11*, were simultaneously detected in the two years and explained the average phenotypic variance of 3.6%, 4.1%, 3.2% and 3.3%, respectively (Table 1; Additional file 3: Figure S2).

For SVN, five QTL were detected on chromosomes 1, 2, 5, and 7, including three (*qSVN1*, *qSVN2* and *qSVN5.2*) detected in 2016 and two (*qSVN5.1* and *qSVN7*) in the two years. The first three QTL accounted for phenotypic variance from 3.5% to 3.6% while the latter two QTL explained the average phenotypic variance of 4.3% and 3.9%, respectively (Table 1; Additional file 3: Figure S2).

For total area of small vascular bundle (SVA), a total of six QTL were detected on chromosomes 2, 8, 11, and 12. Two QTL (*qSVA2.1* and *qSVA11*) were detected in 2015, and accounted for 3.7% and 3.6% of phenotypic variance, respectively. The other four QTL (*qSVA2.2*, *qSVA8.1*, *qSVA8.2* and *qSVA12*) were detected in 2016, and accounted for 3.4% to 3.8% of phenotypic variance (Table 1; Additional file 3: Figure S2).

For SVPA, two QTL, *qSVPA4.1* and *qSVPA4.2* were detected on chromosome 4 in 2016, and accounted for 3.7% and 3.9% of phenotypic variance, respectively (Table 1; Additional file 3: Figure S2).

For xylem area of small vascular bundle (SVXA), four QTL were detected on chromosomes 2, 8, 9 and 11. Of them, two QTL (*qSVXA9* and *qSVXA11*) were detected in 2015 and accounted for 4.4% and 4.5% of phenotypic variance, respectively. Another two QTL (*qSVXA2* and *qSVXA8*) were detected in 2016 and explained 3.5% and 3.4% of phenotypic variance, respectively (Table 1; Additional file 3: Figure S2). There was no any QTL simultaneously detected in the two years.

### Candidate gene analysis

For eight peduncle vascular bundle related traits, we conducted gene-based association and haplotype analysis to identify candidate genes for important QTL identified using the whole population. In addition, considering that among the eight peduncle vascular bundle related traits LVN, SVA, SVXA and SVPA were both significantly different between *indica* and *japonica* in 2015 and 2016 (Additional file 4: Figure S3), we conducted haplotype analysis of targeted genes related to LVN, SVA, SVXA and SVPA in *indica* and *japonica* subpopulations, respectively.

**Fig. 3:**
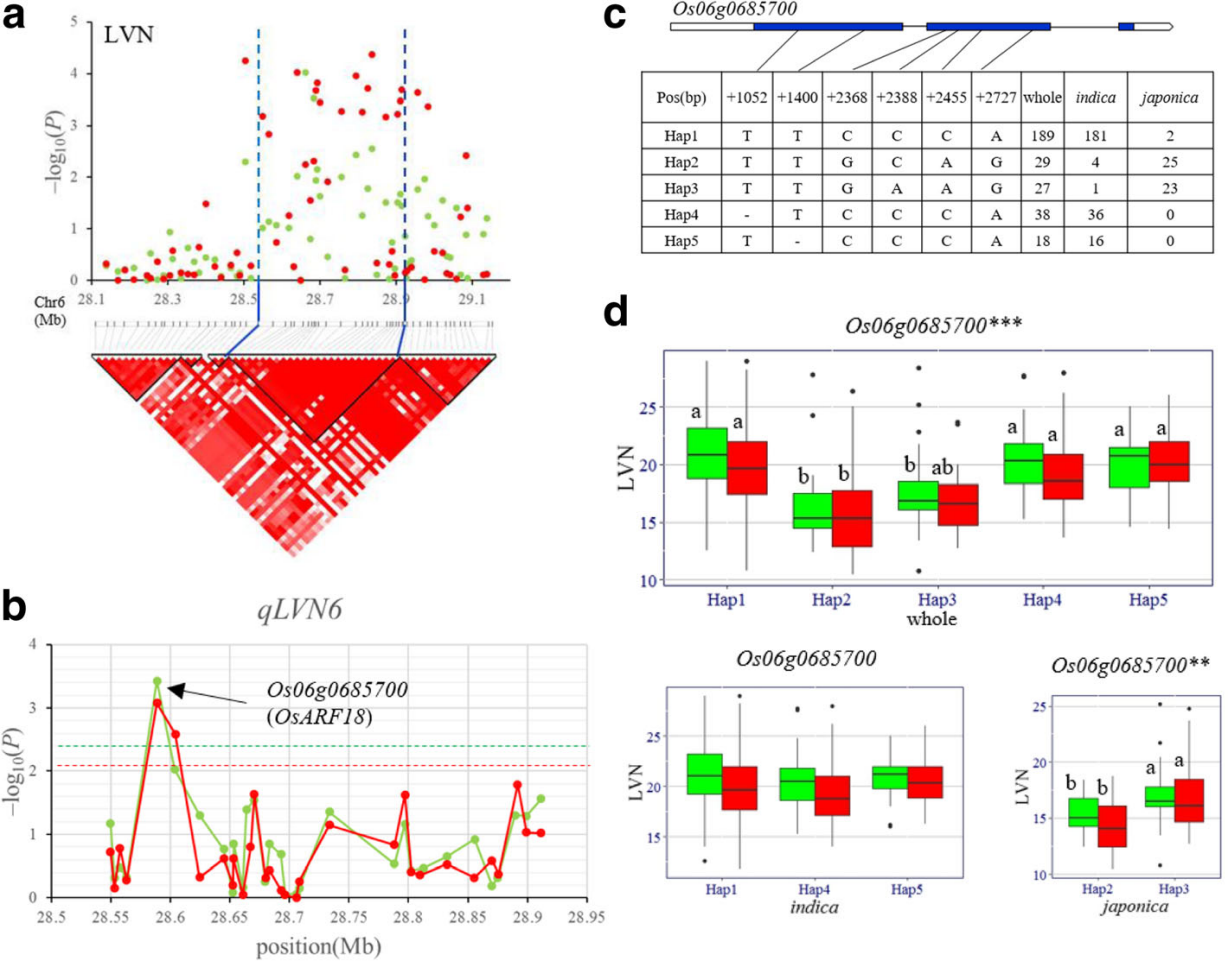
Gene-based association analysis and haplotype analysis of targeted genes related to *qLVN6*. **a** Local Manhattan plot (top) and LD block (bottom) surrounding the peak on chromosome 6. Dashed lines indicate the candidate region for the peak. **b** Gene-based association analysis of targeted genes related to *qLVN6*. Each point is a gene indicated by one SNP having largest –log10 (*P*) value. Dash line show the threshold to determine significant SNP. **c** Exon-intron structure of *Os06g0685700* and DNA polymorphism in that gene. **d** Boxplots for LVN based on the haplotypes (Hap) for *Os06g0685700* in the whole, *indica* and *japonica* populations. The ** and *** suggest significance of ANOVA at *P* < 0.01 and *P* < 0.001, respectively. The letter on histogram (a, and b) indicate multiple comparisons result at the significant level 0.05. Green and red colors indicate in 2015 and 2016, respectively

Using gene-based association analysis and haplotype analysis of candidate gene, six important candidate genes were identified for the four QTL regions distributing on chromosomes 4, 6, 7 and 8, ranging from one to two candidate genes for each QTL. For *qLVN6* in the candidate region of 28.55–28.92 Mb (370 kb) on chromosome 6 (Fig. 3a), 616 non-synonymous SNPs of 52 genes were used for association analysis according to the Rice Genome Annotation Project Database (RAP-DB). The peak SNP was at 28588881 bp on chromosome 6 with –log_10_ (*P*) of 3.43 and 3.08 in 2015 and 2016, respectively. Again, SNPs with –log_10_ (*P*) above the threshold (2.43 in 2015 and 2.08 in 2016) were found to locate in one gene, *Os06g0685700* (Fig. 3b). Significant differences for LVN between different haplotypes were observed at *Os06g0685700* in whole population (Fig. 3c–d).In this study, haplotype was separately analyzed for *indica* and *japonica* varieties owing to the significant differentiation in LVN between the two subspecies. Highly significant differences in LVN were detected between different haplotypes at the candidate gene (*Os06g0685700*) in *japonica*, though there was no significant difference for LVN between different haplotypes in *indica* accessions (Fig. 3c–d).

**Fig. 4:**
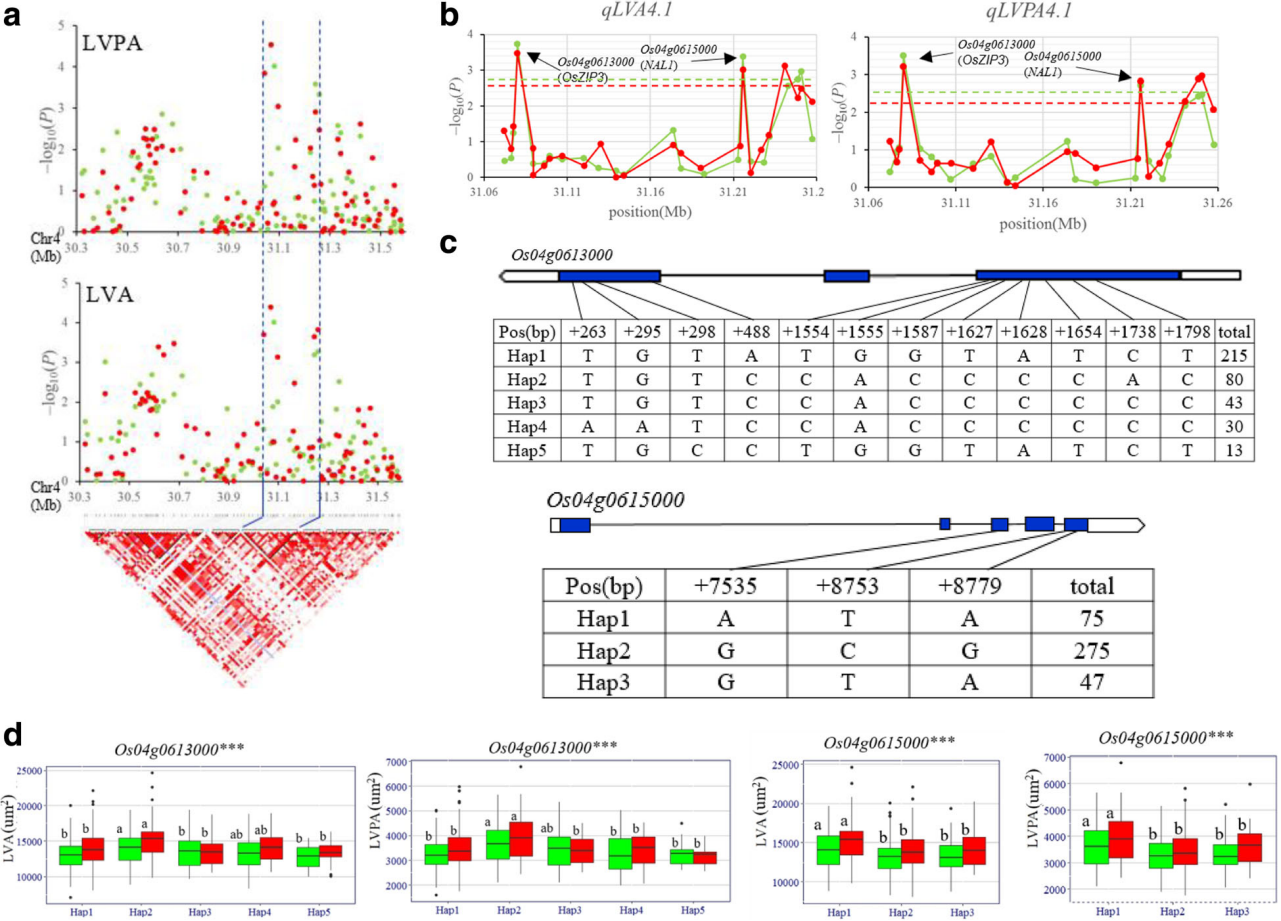
Gene-based association analysis and haplotype analysis of targeted genes related to *qLVA4.1* and *qLVPA4.1*. **a** Local Manhattan plot (top) and LD block (bottom) surrounding the peak on chromosome 4. Dashed lines indicate the candidate region for the peak. **b** Gene-based association analysis of targeted genes related to *qLVA4.1* and *qLVPA4.1*. Each point is a gene indicated by one SNP having largest –log10 (*P*) value. Dash line show the threshold to determine significant SNP. **c** Exon-intron structure of *Os04g0613000* and *Os04g0615000,* and DNA polymorphism in those gene. **d** Boxplots for LVA and LVPA based on the haplotypes (Hap)for *Os04g0613000* and *Os04g0615000*. The *** suggest significance of ANOVA at *P* < 0.001. The letter on histogram (a, and b) indicate multiple comparisons result at the significant level 0.05. Green and red colors indicate in 2015 and 2016, respectively

Similarly, the candidate region was predicted in the region from 31.07 to 31.26 Mb (190 kb) on chromosome 4 harboring *qLVA4.1* and *qLVPA4.1* (Fig. 4a), and 303 non-synonymous SNPs of 30 genes were used for association analysis. The peak SNP was at 31079854 bp with –log_10_ (*P*) of 3.73 (3.47) in 2015 (2016) for LVA, and 3.49 (3.21) in 2015 (2016) for LVPA. Two genes harboring SNPs with –log_10_ (*P*) larger than the thresholds were identified, including *Os04g0613000* and *Os04g0615000* (Fig. 4b). Highly significant differences for *qLVA4.1* and *qLVPA4.1* between different haplotypes were observed at the two genes (*Os04g0613000* and *Os04g0615000*) in whole population (Fig. 4c**–**d). Of the two genes, *Os04g0615000* is identical to *NAL1* (*NARROW LEAF1*), the gene regulating the development of vascular bundle in previously reported (Qi et al. 2008). Three haplotypes of *NAL1* were found and haplotype ATA was associated with significantly larger LVA and LVPA than haplotypes GCG and GTA (Fig. 4c–d).

**Fig. 5:**
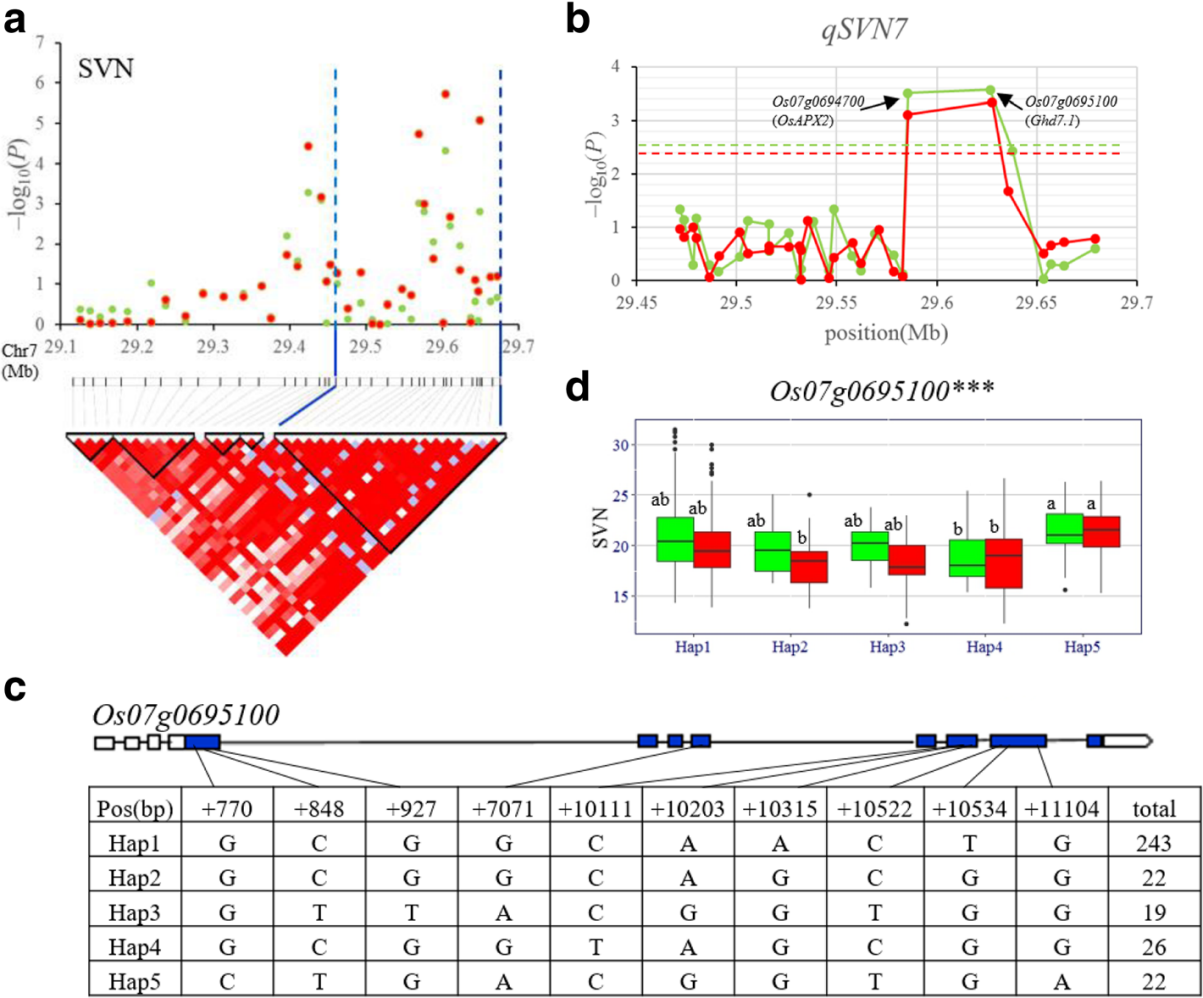
Gene-based association analysis and haplotype analysis of targeted genes related to *qSVN7*. **a** Local Manhattan plot (top) and LD block (bottom) surrounding the peak on chromosome 7. Dashed lines indicate the candidate region for the peak. **b** Gene-based association analysis of targeted genes related to *qSVN7*. Each point is a gene indicated by one SNP having largest –log10 (*P*) value. Dash line show the threshold to determine significant SNP. **c** Exon-intron structure of *Os07g0695100* and DNA polymorphism in that gene. **d** Boxplots for LVN based on the haplotypes (Hap) for *Os07g0695100*. The *** suggest significance of ANOVA at *P* < 0.001. The letter on histogram (**a**, and **b**) indicate multiple comparisons result at the significant level 0.05. Green and red colors indicate in 2015 and 2016, respectively

*QSVN7* was fine-mapped in the region of 29.46–29.67 Mb (210 kb) on chromosome 7 (Fig. 5a), and 353 non-synonymous SNPs of 28 genes were used for association analysis. The peak SNP for *qSVN7* was at 29626817 bp on chromosome 7 with –log_10_ (*P*) of 3.57 and 3.34 in 2015 and 2016, respectively. SNPs with –log_10_ (*P*) above the thresholds were located in the two candidate genes, *Os07g0695100* and *Os07g0694700* (Fig. 5b). No significant haplotypes were found in *Os07g0694700*. Five haplotypes were found for *Os07g0695100*. Haplotypes analysis revealed that significant differences for SVN were observed between haplotypes GCGGTAGCGG and CTGACGGTGA in both years (Fig. 5c–d).

**Fig. 6:**
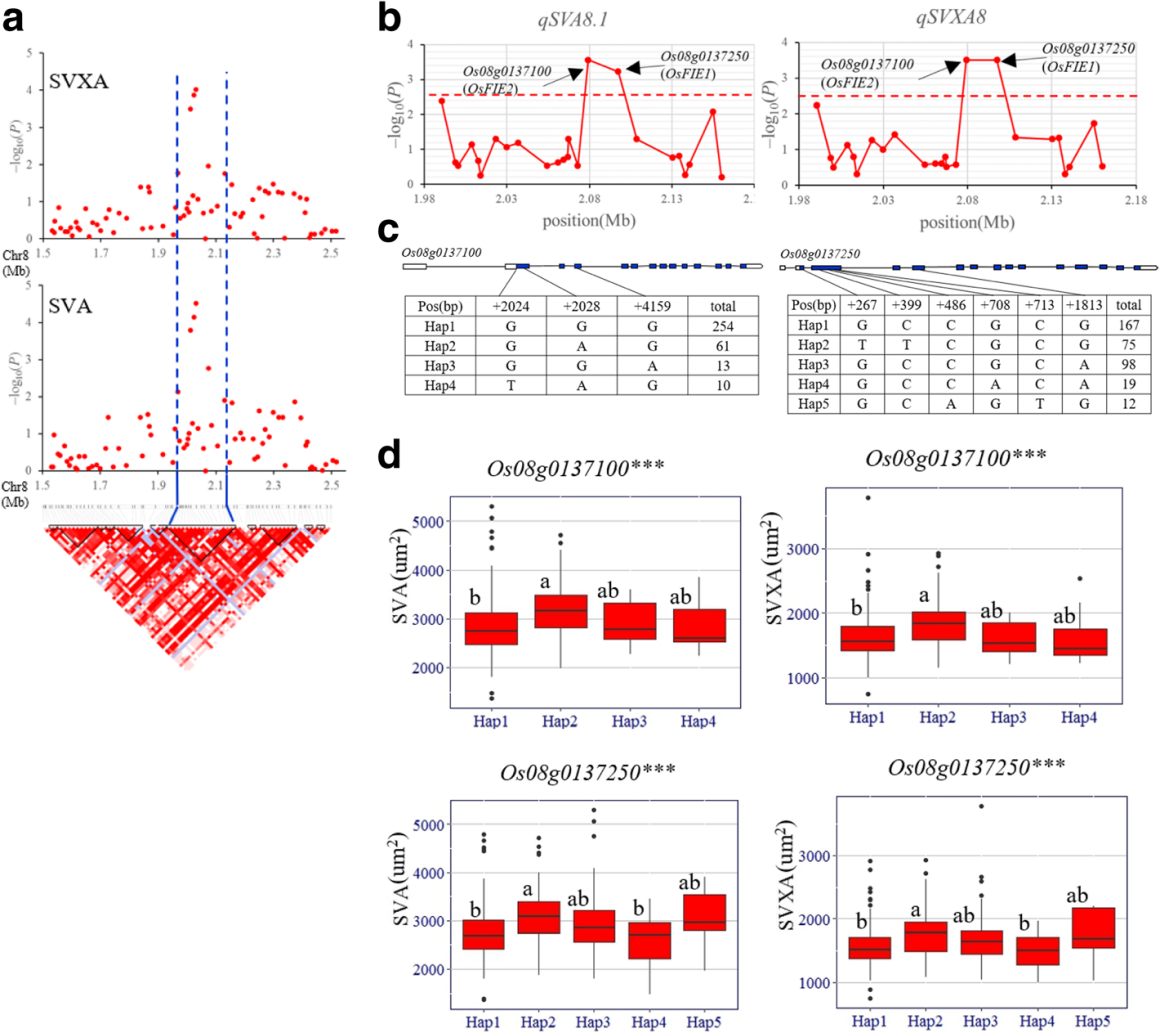
Gene-based association analysis and haplotype analysis of targeted genes related to *qSVA8.1* and *qSVXA8*. **a** Local Manhattan plot (top) and LD block (bottom) surrounding the peak on chromosome 8. Dashed lines indicate the candidate region for the peak. **b** Gene-based association analysis of targeted genes related to *qSVA8.1* and *qSVXA8*. Each point is a gene indicated by one SNP having largest –log10 (*P*) value. Dash line show the threshold to determine significant SNP. **c** Exon-intron structure of *Os08g0137250* and *Os08g0137100,* and DNA polymorphism in those gene. **d** Boxplots for SVA and SVXA based on the haplotypes (Hap) for *Os08g0137250* and *Os08g0137100*. The *** suggest significance of ANOVA at *P* < 0.001. The letter on histogram (a, and b) indicate multiple comparisons result at the significant level 0.05. Green and red colors indicate in 2015 and 2016, respectively

On chromosome 8, a high peak of *qSVA8.1* was found to be overlapped with the peak of *qSVXA8*. We predicted the candidate region from 1.99–2.17 Mb (180 kb) (Fig. 6a), and 277 non-synonymous SNPs of 29 genes were used for association analysis. The peak SNP was at 2079621 bp on chromosome 8 with –log_10_ (*P*) of 3.56 for *qSVA8.1* and 3.50 for *qSVXA8* in 2016. Two genes harboring the SNPs with –log_10_ (*P*) larger than the threshold (2.56 and 2.50 for *qSVA8.1* and *qSVXA8*, respectively) were identified, including *Os08g0137100* and *Os08g0137250* (Fig. 6b). Significant differences were detected among different haplotypes at the two genes in whole population (Fig. 6c–d). However, no significant difference in SVA and SVXA was detected between different haplotypes at the two candidate genes in *indica* subpopulation, and only one prevalent haplotype of each gene was detected in *japonica* subpopulation (Additional file 5: Table S2). Among them, *Os08g0137100* is identical to *OsFIE2*, a gene affecting vascular bundle related traits in previously report (Liu et al. 2016).

## Discussion

### Characteristics of vascular bundle between *Indica* and *Japonica*

*Indica* and *japonica* are the two major types of Asian cultivated rice (*Oryza sativa* L.). There are significant differences between them in many morphological and physiological traits associated with the processes of *indica*-*japonica* differentiation (Morishima and Oka 1981). Among them, the differentiation in vascular bundle system is an important parameter that defines differentiation between the two subspecies. *Indica* varieties tend to have more large vascular bundles in peduncle than *japonica* varieties, and have significant higher ratio of the LVN to the number of primary rachis branches (ranged from 1.3 to 2.2) than *japonica* (nearly1.0) (Fukuyama and Takayama 1995). Our studies showed that LVN in peduncle of *indica* (averaged 21 in 2015 and 19.9 in 2016) was also significantly higher than those of *japonica* (averaged 16.1 in 2015 and 15.5 in 2016) (Additional file 4: Figure S3). In *japonica*-type cultivars, each of the large vascular bundles is directly connected to a primary rachis branch (PRB), resulting almost in same quantity of LVN in the peduncle and number of PRBs. On the other hand, in *indica*-type some large vascular bundles are directly connected to the secondary rachis branches due to surplus large vascular bundles (Fukushima and Akita 1997; Terao et al. 2010). The different vascular bundle characteristics between *indica* and *japonica* probably explained inherently shorter grain development phase (Osada et al. 1983) or shorter grain filling period (Nagato and Chaudhry 1969) in *indica* than *japonica* resulting from more rapid multiplication of endosperm cells (Nagato and Chaudhry 1969) and dominant transportation efficiency of photosynthetic products (Weng and Chen 1987) in *indica*. Rice grain yield is highly positively significantly correlated with spikelet number on first rachis branches especially secondary rachis branches. So, genetic mechanisms and their genetic relationships between the number of rachis branches and vascular bundle related traits in rice need further explored.

### Candidate gene identification of the important QTL

Cloning QTL affecting complex traits has been a major challenge to plant geneticists and molecular biologists since the classical strategy using map-based cloning is extremely troublesome and time-consuming. Using GWAS and haplotype analysis of candidate genes, we were able to shortlist six candidate genes governing the four important QTL affecting vascular bundles related traits in the peduncle.

In the region of 31.07–31.26 Mb on chromosome 4, a QTL cluster (*qLVA4.1* and *qLVPA4.1*) was detected, containing two candidate genes. One of them was *NAL1* (*Os04g0615000*) regulating the development of vascular bundle which may be involved in polar auxin transport (Qi et al. 2008) and contributed to grain yield (Fujita et al. 2013; Xu et al. 2015). Culms of *nal1* showed a defective vascular system, in which the number and distribution pattern of vascular bundles was altered (Qi et al. 2008). Additionally, the Takanari allele of *NAL1*, which was found to be a partial loss-of-function allele of *NAL1*, increased mesophyll cell number between vascular bundles (Takai et al. 2013). Haplotypes analysis revealed haplotype ATA was associated with significantly larger LVA and LVPA than haplotypes GCG and GTA (Fig. 4c–d). Further, we found one nucleotide variation located in the third exon which results in the substitution of a histidine (CAT) in the trypsin-like serine and cysteine protease domain of the large LVA and LVPA by an arginine (CGT) in the small LVA and LVPA (Additional file 6: Fig. S4). And an allele variation produced less panicles per plant, more spikelet numbers and more width leaf blades than varieties containing G allele (Yano et al. 2016). Taguchi-Shiobara et al. (2015) reported that the flag leaf width of transgenic plants of *NAL1* varied depending on the ratio of A allele to G allele. Thus, we presumed that the nucleotide variation (A/G) in the third exon exerted a critical function on determining panicles per plant, spikelet numbers per panicle, flag leaf width and area of large vascular bundle in the peduncle.

In the present study, two QTL (*qLVN6* and *qSVN7*) were detected in both two years. The *qLVN6* affecting LVN was identified in the region of 28.55–28.92 Mb on chromosome 6.The only candidate gene for *qLVN6* was *OsARF18* (*Os06g0685700*), a similar to auxin response factor gene. The deregulation of the *OsmiR160* target gene *OsARF18* leads to rice abnormal growth and development, including dwarf stature, rolled leaves, and small seeds through affecting the auxin signaling (Huang et al. 2016). Compared with wild type, numbers of bulliform cell and total abaxial epidermal cells between two vascular bundles were significantly reduced in *mOsARF18* leave (Huang et al. 2016). A number of studies have shown that polar auxin transport is required for continuous vascular pattern formation and establishment of procambial strands (Mattsson et al. 2003; Scarpella et al. 2006; Berleth et al. 2007). The classic auxin signal flow canalization hypothesis predicts that the distribution of auxin is narrowed down from a wide field of cells to a subset of cells with high auxin transport, which then become the site of the procambium (Sachs 1981). Then gene function in *PINT*, *MP* and *ATHB8*, which is a positive feedback loop of auxin–*MP*–*ATHB8*–*PIN1*, confirms the hypothesis (Paciorek et al. 2005; Scarpella et al. 2006; Donner et al. 2009). In addition, *AVB* gene affecting vascular bundles involved in procambium establishment following auxin signaling in lateral primordial (Ma et al. 2017). Previously, researches considered that *NAL1* (*Os04g0615000*) regulating the development of vascular bundle played its role in leaf morphogenesis by regulating polar auxin transport, and plays a regulatory role in the development of plant type in rice (Qi et al. 2008; Fujita et al. 2013). Therefore, *OsARF18* (*Os06g0685700*) is considered as a possible candidate gene of *qLVN6*. Transgenic experiments are under way to verify the functionalities of the candidate genes. The only candidate gene for *qSVN7*, *Os07g0695100* (*Ghd7.1*) was a major QTL with pleiotropic effects on grain number per panicle, plant height, and heading date in rice (*Oryza sativa* L.) in previously report (Liu et al. 2013; Gao et al. 2014). In this study, compared with haplotype GCGGTAGCGG, haplotype CTGACGGTGA delayed heading and increased SVN, grain number per panicle and plant height in 2016 Sanya (Additional file 7: Figure S5), hinting that *Ghd7.1* probably affects vascular bundle in rice. However and so far there has been no reported *Ghd7.1* gene relevant to vascular bundle. Thus, as to whether *Ghd7.1* is candidate gene for *qSVN7*, it must be verified using reverse genetic approaches.

Of the two candidate genes for *qSVA8.1*, *OsFIE2* (*Os08g0137100*) belongs to the PcG protein family and is the most likely candidate gene. Compared to wild-type, the number of large and small vascular bundles decreased in second internode of *osfie2*–1 stem, as well as cell number and cell size in spikelet hulls (Liu et al. 2016). However, no significant difference was detected between different haplotypes at *Os08g0137100* in *indica* subpopulation, and only one haplotype was observed in *japonica* subpopulation (Additional file 5: Table S2), though there were significant differences for SVA and SVXA between different haplotypes in whole population (Fig. 6d), suggesting that *indica*-*japonica* differentiation probably led to significant phenotypic differences in SVA and SVXA between different haplotypes. Thus, it must be further studies that *OsFIE2* is candidate gene for *qSVA8.1* or evolution gene of *indica* and *japonica*.

### Application in Rice breeding for improved vascular bundle system

The grain yield of crop is highly dependent on the source-sink relationship in which grain or kernel is the photosynthetic sink while the leaves are the primary source. The vascular bundle is the transport system that links source and sink, and it determines how plants efficiently transport photosynthetic products, mineral nutrients and water from source to sink (Housley and Peterson 1982). Efficient transport of assimilates from leaves and stems to developing spikelets is required for better grain filling and high yield. *SPIKE* gene increases grain yield of *indica* rice through enlarging sink size, source size, and translocation capacity. Compared with that of IR64 isogenic control, the grain fertility of NIL-*SPIKE* was improved, presumably owing to a strengthening of assimilate supply to the larger number of spikelets by an increase in vascular bundle number of panicle neck (Fujita et al. 2013). *APO1* gene controlling the number of primary rachis branches also controls the vascular bundle formation and hence is responsible to increase the harvest index and grain yield in rice (Terao et al. 2010). The effect on vascular bundle system development regulated by the *HI1* allele of *APO1* limited reduction in ripening percentage (Terao et al. 2010). In a word, the capacity of transporting assimilates from source to sink could limit grain filling (Ashraf et al. 1994). What rice breeders concern is how to develop new variety with an advanced vascular bundle system and a strong ability to transport assimilates from source to sink.

Our results suggested that in the region of 28.55–28.92 Mb on chromosome 6, a QTL (*qLVN6*) was detected in both two years affecting LVN. According to gene function annotation, *Os06g0685700*, a similar to auxin response factor gene is considered as the most likely candidate gene of *qLVN6*. Haplotype analysis revealed that significant differences for LVN were between different haplotypes at the gene in *japonica* accessions. Two tropical *japonica* varieties, PLUS (averaged 20.4 in 2015 and 23.5 in 2016) and ITA 221 (averaged 21.7 in 2015 and 23.7 in 2016) with more LVNs were found in this panel. Haplotype TTGAAG of the *Os06g0685700* from the two *japonica* accessions exhibited additive effect for increased number of large vascular bundles in peduncle in *japonica* and this favorable QTL allele would be introgressed into *indica* and other *japonica* varieties with superior agronomic traits for improved large vascular bundle by marker-assisted selection (MAS). Specifically, for example, hybrid between *indica* and *japonica* holds large panicle with more primary rachis branches especially secondary rachis branches but its grain yield is seriously limited by insufficient grain filling due to disharmony of source, sink and flow of assimilates. So, it is possible to improve grain filling of hybrid between two subspecies by reasonably deploying favorable alleles of QTL for peduncle vascular bundle related traits in rice as indicated in this study.

## Conclusion

We identified 48 QTL for eight peduncle vascular bundle related traits via GWAS. A total of six candidate genes of four important QTL regions, including the cloned gene (*NAL1*), were identified by determining linkage disequilibrium blocks associated with significant SNPs, and haplotype analyses. In addition, the most likely candidate genes for three new QTL (*qLVN6*, *qSVN7*, and *qSVA8.1*) were identified based on functional annotation. The results will enhance our knowledge of the genetic basis of vascular bundle traits in rice, facilitate exploring the molecular mechanisms underlying the traits, and provide valuable information for improving rice vascular bundle system by MAS breeding.

## Methods

### Plant materials

The 423 worldwide accessions were selected from 3 K RGP (Zheng et al. 2015). This panel consisted of seven types, including *indica* (301), *temperate japonica* (13), *tropical japonica* (57), admixed *japonica* (9), admixed type (17), *aus*/*boro* (16), and *basmati*/*sadri* (10) (Additional file 2: Table S1).

### Phenotypic investigation

All of these accessions were grown in Sanya (18.3^o^N, 109.3°E) during Dec 2014–April 2015 and Dec 2015–April 2016 and each accession was planted in two rows with 10 individuals each row at a spacing of 25 cm between rows and 17 cm between plants with two replications for each accession. The field management followed the farmers’ standard management practices. In order to minimize flowering time effects experiment-wise, batch sampling were performed based on heading date of each accession. At full heading stage, five uniform plants in the middle of each plot were selected. The transverse section of stem were made at uniformly 2 cm above the neck-panicle node and kept in formalin-acetic-alcohol (FAA) fixative solution. After safranin O staining, vascular bundle related traits in the peduncle, including the number of large vascular bundle (LVN), the total area of large vascular bundle (LVA, um^2^), the phloem area of large vascular bundle (LVPA, um^2^), the xylem area of large vascular bundle (LVXA, um^2^), the number of small vascular bundle (SVN), the total area of small vascular bundle (SVA, um^2^), the phloem area of small vascular bundle (SVPA, um^2^) and the xylem area of small vascular bundle (SVXA, um^2^) (Additional file 8: Figure S6), were observed and measured using a microscopy (ZEISS AXIO, Germany). The average trait value of each accession was used in data analyses of GWAS.

### Genotyping

The 27 K SNP genotype data of the 423 accessions was generated from the 3 K RGP (Zheng et al. 2015). For those SNPs, only the alleles of highest frequency in the 423 panels were retained and other alleles of low frequency were considered missing. The heterozygous alleles were also eliminated. SNPs with missing rate over 20% and minor allele frequency (MAF) less than 5% were removed. Finally, a total of 26,097 SNPs were used in the GWAS.

### Population structure and kinship

Principal component (PC) and population structure (Q) were applied to infer population structure. For the 26,097 SNP, we further removed SNP loci with missing rate over 10% and MAF less than 0.10. Then, 6601 evenly distributed SNPs with average marker spacing around 50 kb were sampled to calculate population structure (Q). A model based Bayesian clustering analysis method implemented in STRUCTURE software version 2.3.4 (Pritchard et al. 2000) was used. The program was run with the following parameters: k, the number of groups in the panel varying from 1 to 7; 10 runs each k value; for each run, 10,000 burn in iterations followed by 10,000 MCMC (Markov Chain Monte Carlo) iterations. A principal component analysis (PCA) was performed the efficient mixed-model association (EMMA) method in the Genome Association and Prediction Integrated Tool (GAPIT) R package (Lipka et al. 2012) to examine the population structure. The K matrix (kinship matrix) was obtained from the results of the relatedness analysis using the EMMA method in GAPIT, a package of R software (Lipka et al. 2012). The PCA and K matrix were used in the following association analysis.

### GWAS and identification of candidate genes

We performed a GWAS to excavate the SNPs significant associations with all measured traits using 26,097 SNPs and the mean trait values of the 423 accessions. In this study, the model of mixed linear (MLM), PCA + K, was used in the association analysis. And we performed the study by the software GAPIT. The MLM includes both fixed and random effects. Including individuals as random effects gave an MLM the ability to incorporate information about relationships among individuals. This information about relationships was conveyed through the KI matrix, which was used in an MLM as the variance-covariance matrix between the individuals. Then a genetic marker-based KI matrix was used jointly with PCA (Yu et al. 2006). After the GWAS, SNPs affecting the measured traits were claimed when the test statistics reached *P* < 1.0 × 10^− 4^ in at least one of the two years.

Gene-based association analysis was carried out to detect candidate genes for important QTL. Here, QTL regions meeting at least one of the following criteria were considered as important: (1) consistently identified in both two years; (2) affecting more than one trait; and/or (3) close to previously reported cloned genes or fine mapped QTL. The following five steps were conducted to identify candidate genes for important QTL identified. Firstly, the LD block was analyzed using the software “Haploview v4.2” (Barrett et al. 2005). A LD block was created when the upper 95% confidence bounds of D’ value exceeded 0.98 and the lower bounds exceeded 0.70 (Gabriel et al. 2002). The LD block where the significant trait-associated SNPs were situated was defined as the candidate gene regions. We can find all the genes located in the candidate regions for each important QTL from the Rice Annotation Project Database (RAP-DB).Secondly, all available non-synonymous SNPs located inside of these genes were searched from 32 M SNPs data generated from 3 K RGP in the Rice SNP-Seek Database (Alexandrov et al. 2015). Thirdly, the SNPs with minor allele frequency less than 0.05 and/or missing rate over 20% were removed and remaining the high quality SNPs inside of these candidate genes of each important QTL were used to perform gene-based association analyses through MLM using the PCA and K applied in GWAS. The threshold was defined as –log_10_ (*P*) of the peak SNP of the detected QTL minus 1 (Wang et al. 2017; Zhang et al. 2017). Fourthly, haplotype analysis were carried out for the candidate genes in each important QTL region using all non-synonymous SNPs located inside of the gene CDS region. Finally, candidate genes were determined by testing the significant differences among major haplotypes (containing more than 10 samples) for each important QTL through analysis of variance (ANOVA).

## Additional files

Additional file 1: **Figure S1.** Distribution of SNP markers on chromosomes. The colors show the number of SNPs within 1 Mb window size. (PPT 159 kb)

Additional file 2: **Table S1.** List of the 423 accessions including country of origin, subpopulation (*indica*, *temperate japonica*, *tropical japonica*, admixed *japonica*, admixed type, *aus*/*boro*, and *basmati*/*sadri*), population structure (Q), PC score, vascular bundle related traits measured in 2015 and 2016, and heading date measured in 2016. (XLSX 115 kb)

Additional file 3: **Figure S2.** Genome-wide association results for 8 vascular bundle related traits. Manhattan plots (left) and quantile-quantile plots (right) associated with LVN (a), LVA (b), LVPA (c), LVXA (d), SVN (e), SVA (f), SVPA (g), and SVXA (h) in 423 accessions in 2015 and 2016. For the Manhattan plots, −log_10_ *P*-values from a genome-wide scan were plotted against the position of the SNPs on each of 12 chromosomes and the horizontal grey dashed lines show the suggestive threshold (*P* = 1.0 × 10^− 4^). For the quantile-quantile plots, the horizontal axes indicate the –log_10_ -transformed expected *P* values, and the vertical axes indicate the –log_10_-transformed observed *P*-values. Arrows indicate QTL overlapping with the published QTL. (PPT 1286 kb)

Additional file 4: **Figure S3.** Comparisons of eight vascular bundle traits between *indica* and *japonica* subpopulations. Blue and orange bar indicate *indica* and *japonica*, respectively. All data are presented as the mean ± SD. *, *P* < 0.05 and ***, *P* < 0.001. (PPT 190 kb)

Additional file 5: **Table S2.** Haplotype analysis of the targeted genes related to *qSVA8.1* and *qSVXA8* in the whole population, *indica* and *japonica* subpopulations, respectively. The letters (a, and b) indicate multiple comparison results at the significant level 0.05. (XLSX 10 kb)

Additional file 6: **Figure S4.** Comparison of LVA and LVPA between A and G alleles located in the third exon of *Os04g0615000* (*NAL1*) gene. The letters on histogram (a, and b) indicate multiple comparisons result at the significant level 0.05. The value on the histogram is the number of individuals of each allele. (PPT 217 kb)

Additional file 7: **Figure S5.** Comparisons of heading date, grain number per panicle and plant height among different haplotypes of *Ghd7.1* gene in 2016 Sanya. The letters on histogram (a, b and c) indicate multiple comparison results at the significant level 0.05. The value on the histogram is the number of individuals of each haplotype. (PPT 218 kb)

Additional file 8: **Figure S6.** Cross-sections of large and small vascular bundle in peduncle stained with safranin O staining. P, phloem; XP, xylem parenchyma; PX, protoxylem element; MX, metaxylem element; PXL, protoxylem lacuna. (PPT 699 kb)

## Abbreviations

3 K RGP: 3 K Rice Genome Project
ANOVA: Analysis of variance
EMMA: Efficient mixed-model association
FAA: Formalin-acetic-alcohol
GAPIT: Genome association and prediction integrated tool
GWAS: Genome-wide association study
KI: Kinship
LD: Linkage disequilibrium
LVA: Total area of large vascular bundle
LVN: Number of large vascular bundle
LVPA: Phloem area of large vascular bundle
LVXA: Xylem area of large vascular bundle
MAF: Minor allele frequency
MAS: Marker-assisted selection
MLM: Model of mixed linear
PCA: Principal component analysis
PRB: Primary rachis branch
QTL: Quantitative trait loci/locus
RAP-DB: Rice annotation project database
SNP: Single-nucleotide polymorphism
SVA: Total area of small vascular bundle
SVN: Number of small vascular bundle
SVPA: Phloem area of small vascular bundle
SVXA: Xylem area of small vascular bundle
