# Supplementary figures and images for: QTL mapping and candidate gene analysis of peduncle vascular bundle related traits in rice by genome-wide association study
Source: Rice (N Y). 2018 Mar 6;11:13. doi: 10.1186/s12284-018-0204-7 (PMC5840110; doi:10.1186/s12284-018-0204-7)

## Slide 1
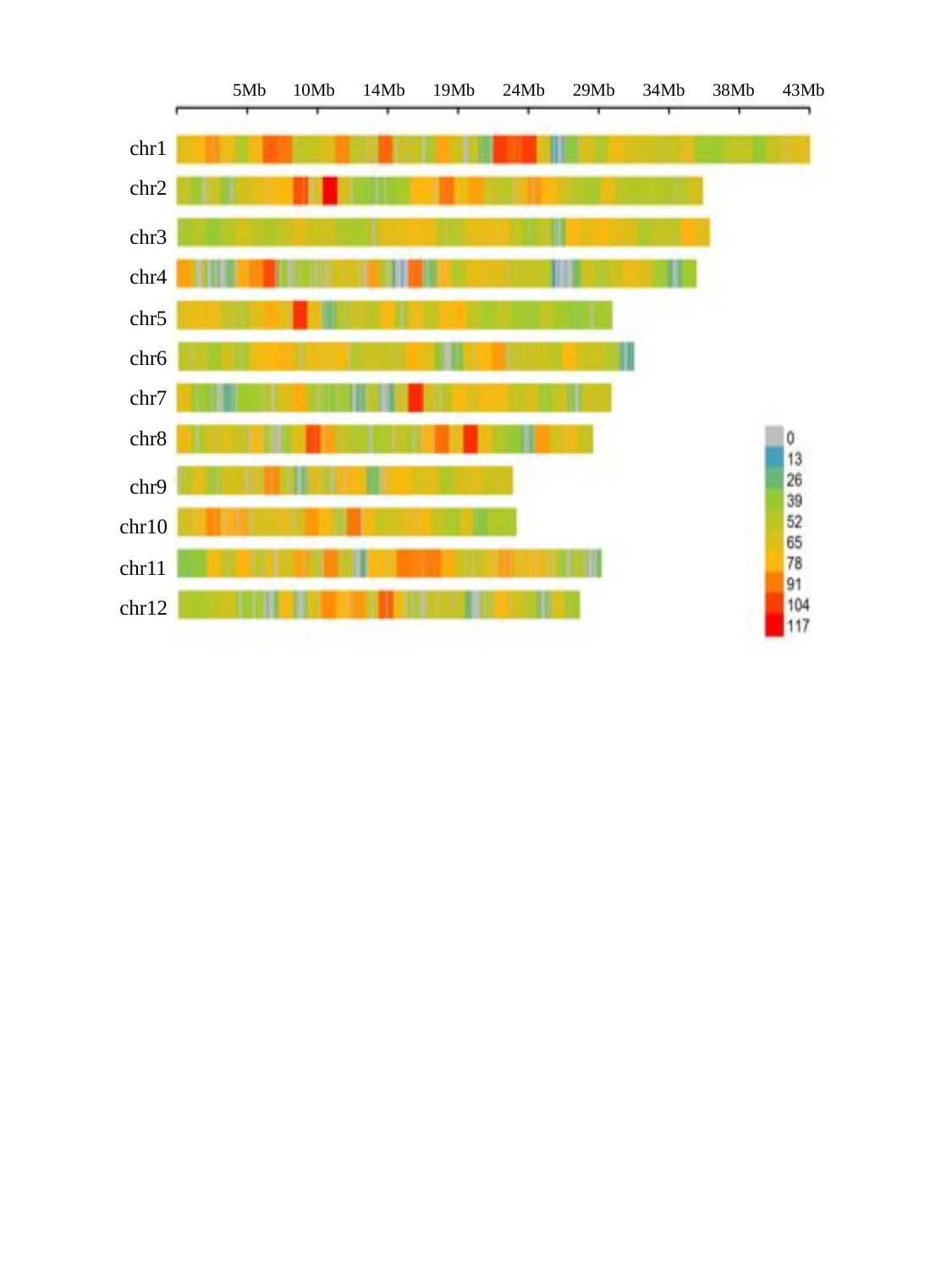

5Mb
10Mb
14Mb
19Mb
24Mb
29Mb
34Mb
38Mb
43Mb
chr1
chr2
chr3
chr4
chr5
chr6
chr7
chr8
chr9
chr10
chr11
chr12

Supplement: Supplementary file 1 — Figure S1. Distribution of SNP markers on chromosomes. The colors show the number of SNPs within 1 Mb window size. (PPT 159 kb) [file 12284_2018_204_MOESM1_ESM.ppt]

## Slide 1
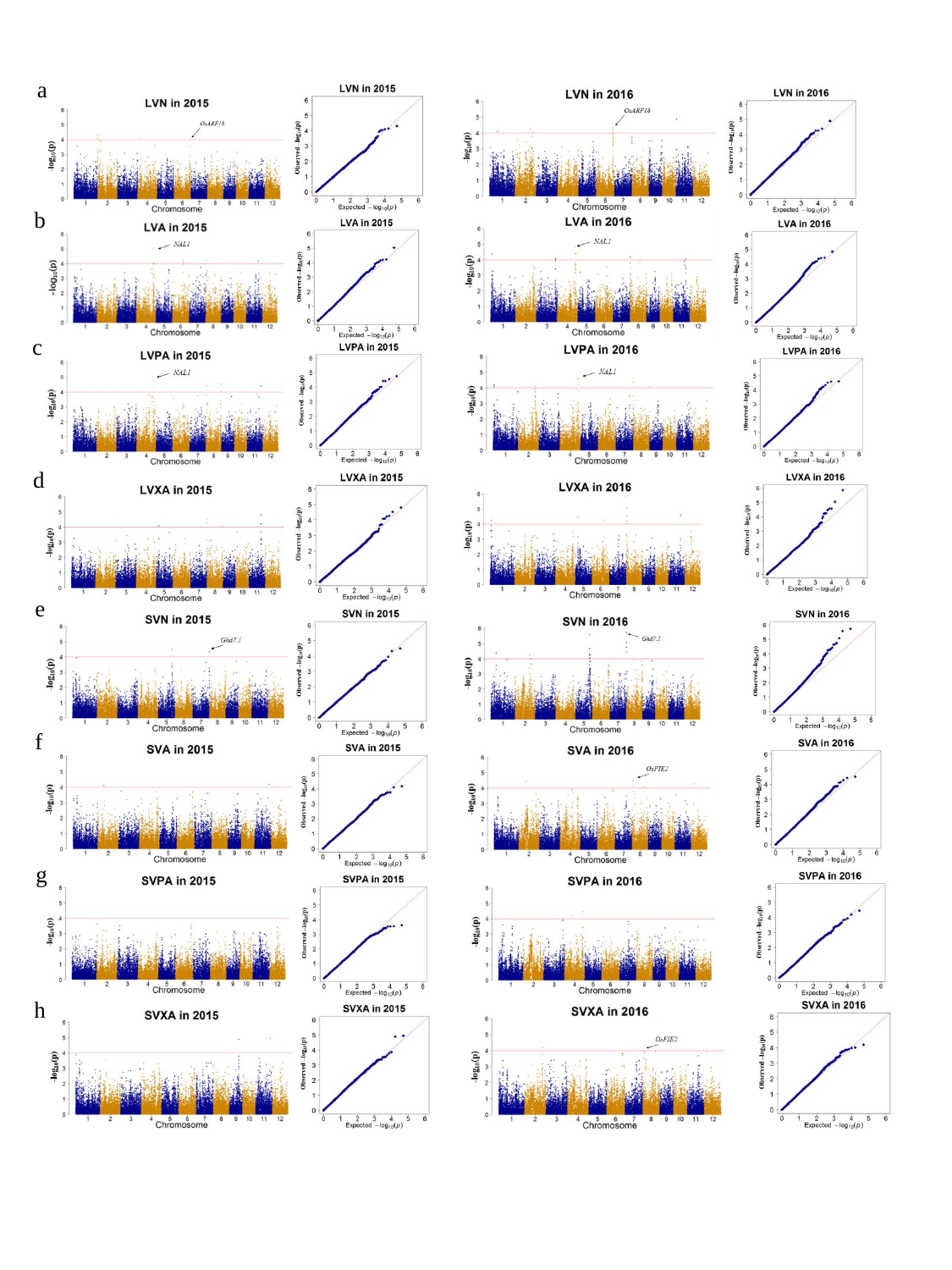

a
b
c
d
e
f
g
h

Supplement: Supplementary file 3 — Figure S2. Genome-wide association results for 8 vascular bundle related traits. Manhattan plots (left) and quantile-quantile plots (right) associated with LVN (a), LVA (b), LVPA (c), LVXA (d), SVN (e), SVA (f), SVPA (g), and SVXA (h) in 423 accessions in 2015 and 2016. For the Manhattan plots, −log10 P-values from a genome-wide scan were plotted against the position of the SNPs on each of 12 chromosomes and the horizontal grey dashed lines show the suggestive threshold (P = 1.0 × 10− 4). For the quantile-quantile plots, the horizontal axes indicate the –log10 -transformed expected P values, and the vertical axes indicate the –log10-transformed observed P-values. Arrows indicate QTL overlapping with the published QTL. (PPT 1286 kb) [file 12284_2018_204_MOESM3_ESM.ppt]

## Slide 1
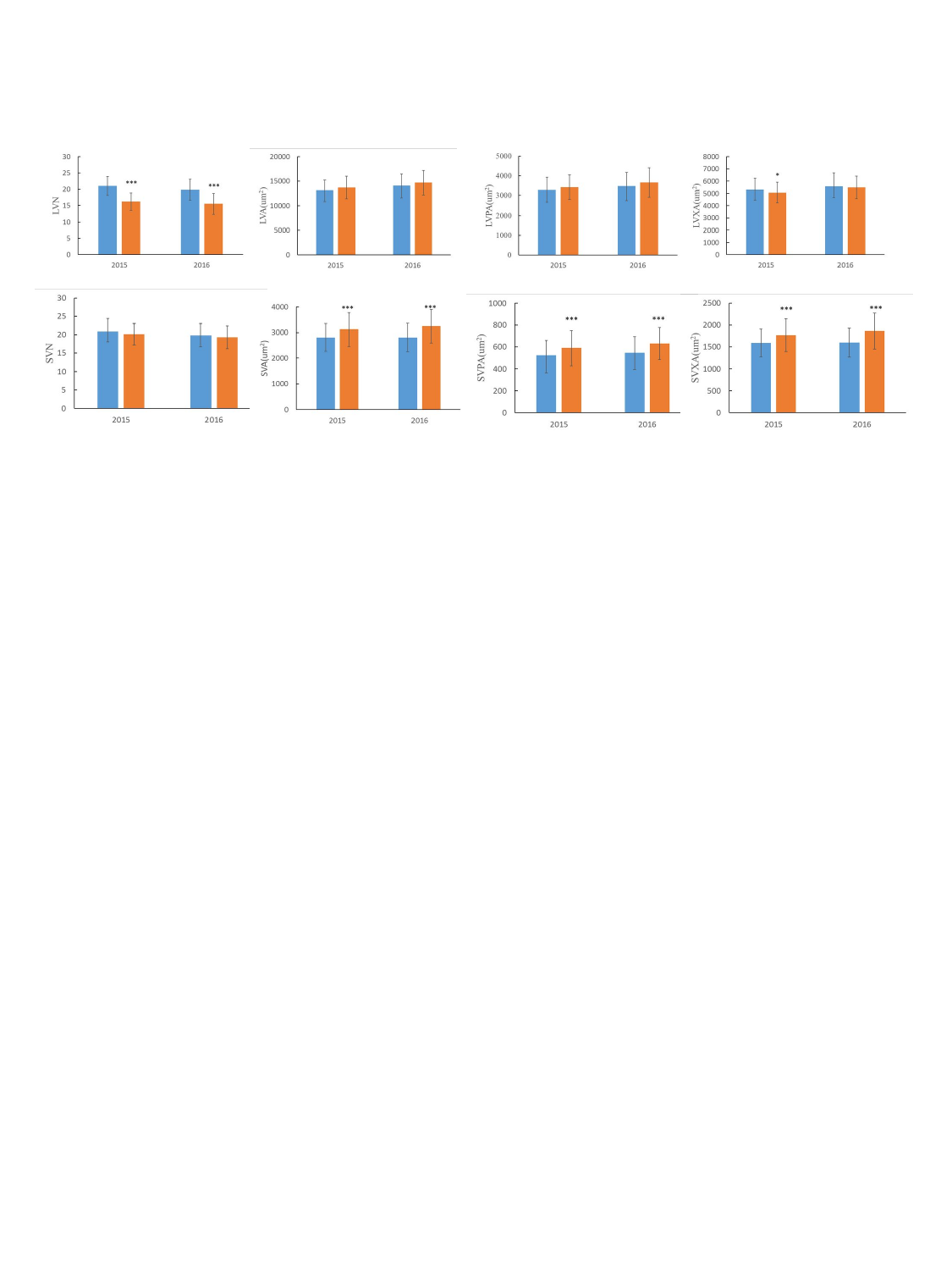

Supplement: Supplementary file 4 — Figure S3. Comparisons of eight vascular bundle traits between indica and japonica subpopulations. Blue and orange bar indicate indica and japonica, respectively. All data are presented as the mean ± SD. *, P < 0.05 and ***, P < 0.001. (PPT 190 kb) [file 12284_2018_204_MOESM4_ESM.ppt]

## Slide 1
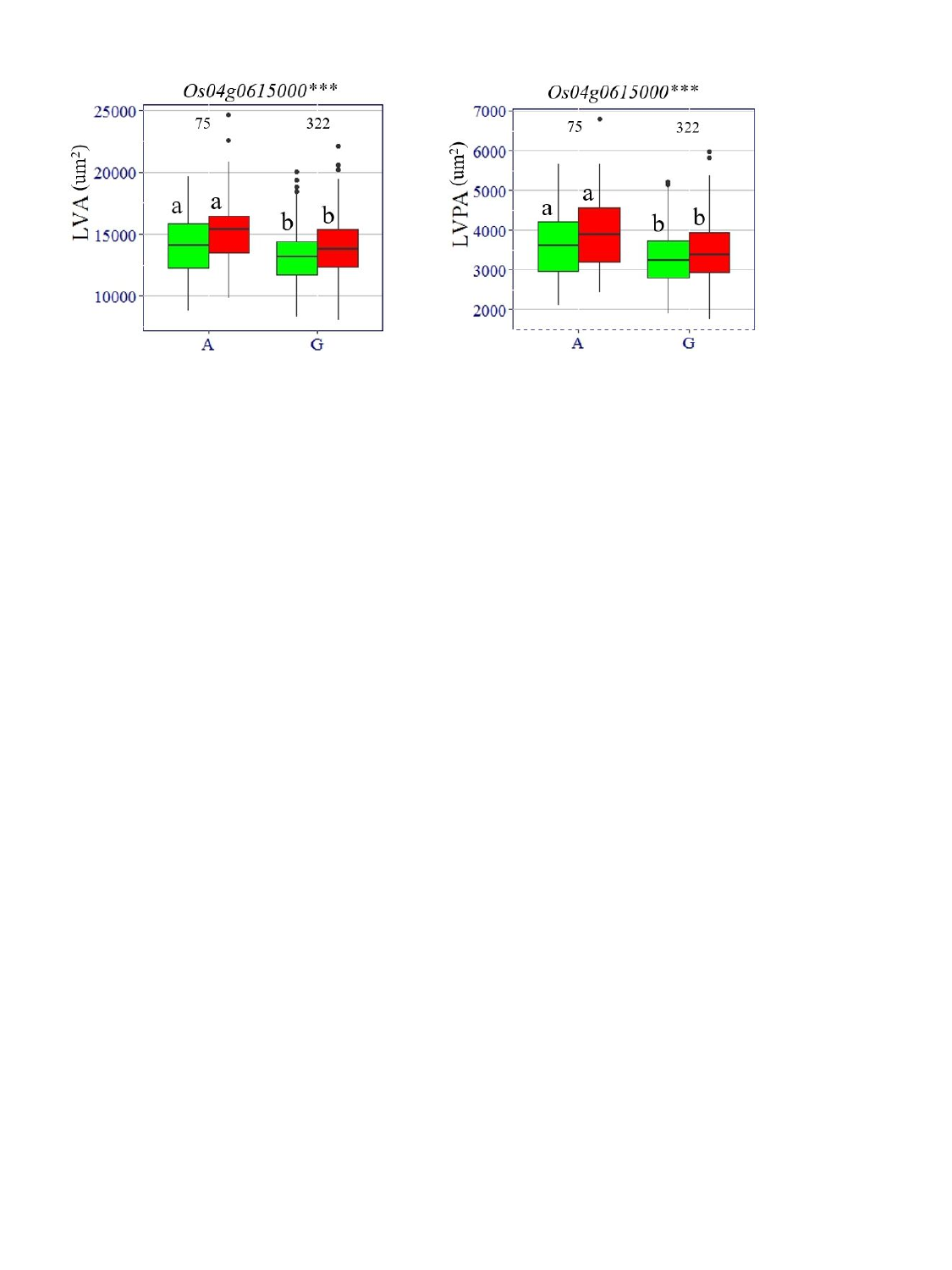

Supplement: Supplementary file 6 — Figure S4. Comparison of LVA and LVPA between A and G alleles located in the third exon of Os04g0615000 (NAL1) gene. The letters on histogram (a, and b) indicate multiple comparisons result at the significant level 0.05. The value on the histogram is the number of individuals of each allele. (PPT 217 kb) [file 12284_2018_204_MOESM6_ESM.ppt]

## Slide 1
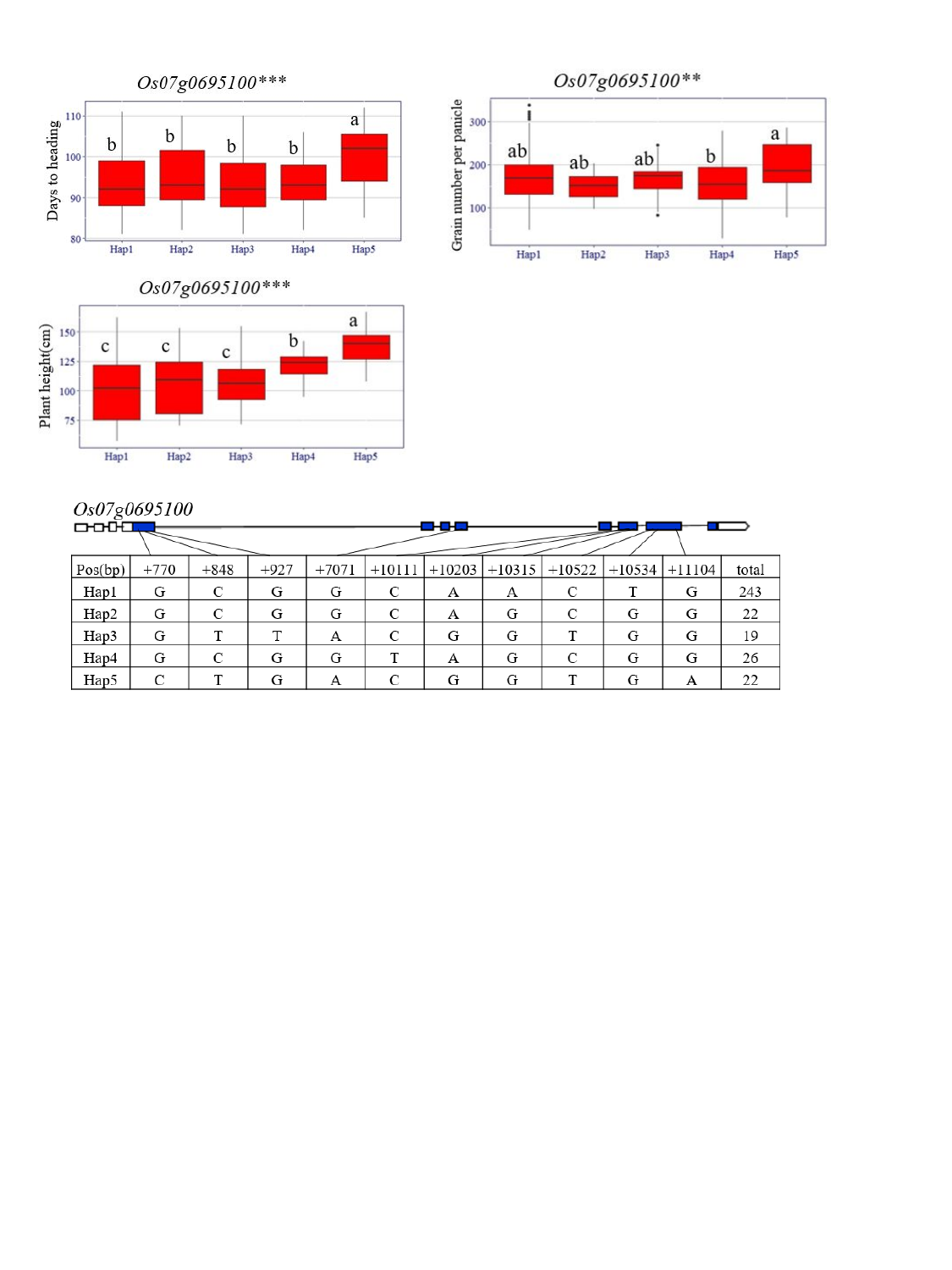

Supplement: Supplementary file 7 — Figure S5. Comparisons of heading date, grain number per panicle and plant height among different haplotypes of Ghd7.1 gene in 2016 Sanya. The letters on histogram (a, b and c) indicate multiple comparison results at the significant level 0.05. The value on the histogram is the number of individuals of each haplotype. (PPT 218 kb) [file 12284_2018_204_MOESM7_ESM.ppt]

## Slide 1
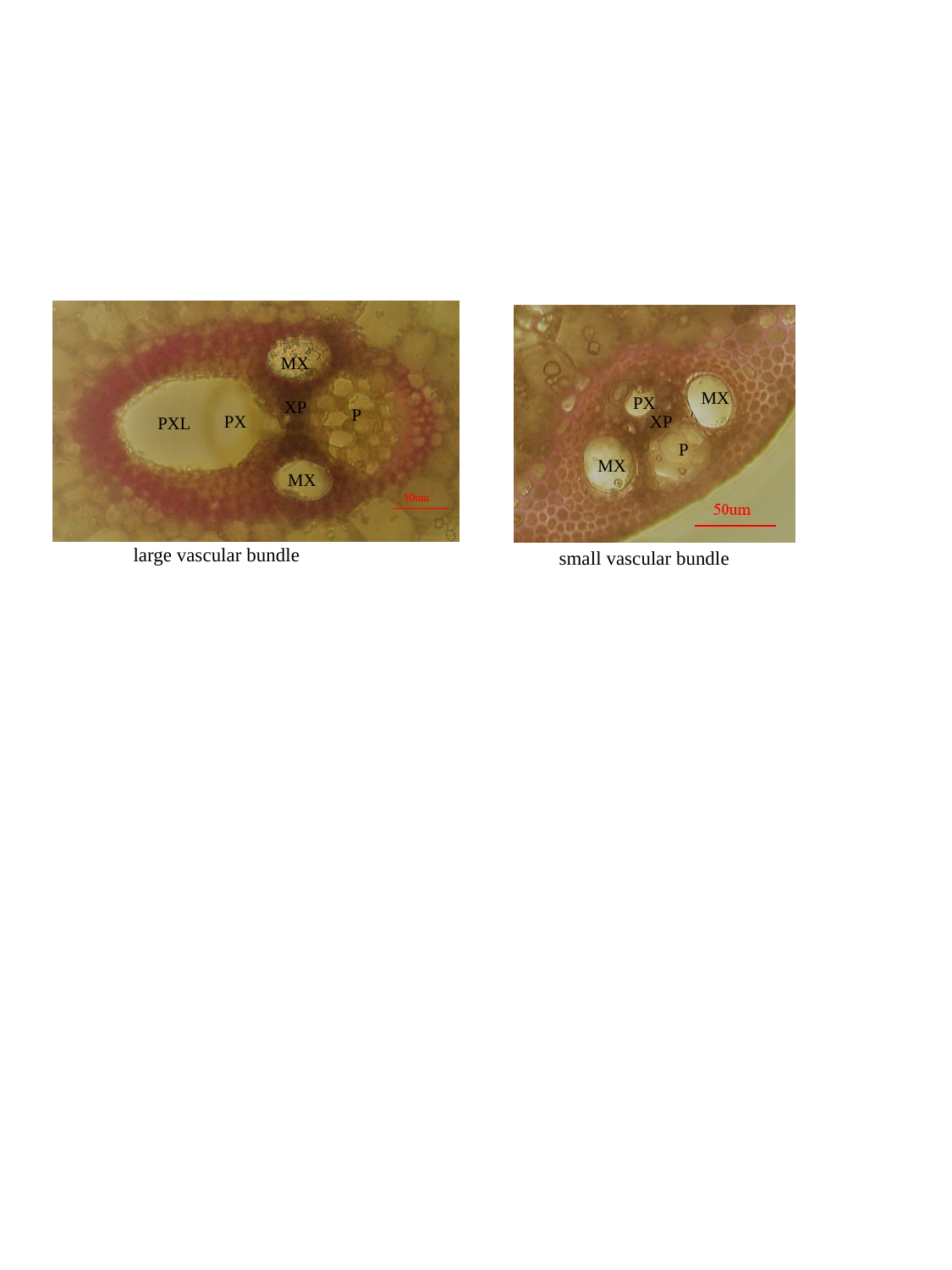

MX
MX
PX
XP
P
PX
XP
PXL
P
MX
MX
large vascular bundle
small vascular bundle

Supplement: Supplementary file 8 — Figure S6. Cross-sections of large and small vascular bundle in peduncle stained with safranin O staining. P, phloem; XP, xylem parenchyma; PX, protoxylem element; MX, metaxylem element; PXL, protoxylem lacuna. (PPT 699 kb) [file 12284_2018_204_MOESM8_ESM.ppt]
